# Supplementary material for: Testing “efficient supply chain propositions” using topological characterization of the global supply chain network
Source: PLoS One. 2020 Oct 1;15(10):e0239669. doi: 10.1371/journal.pone.0239669 (PMC7529254; doi:10.1371/journal.pone.0239669)
Supplement: S1 Appendix — (PDF) [file pone.0239669.s001.pdf]

Appendix S1: Testing "efficient supply chain propositions" using topological characterization of the global supply chain network

- Table S1 represents the code of countries, firm distribution in different countries, total revenue of the firms in each country and countries' GDP (current US$) for the year 2017.
- Table S2 represents the industrial sectors and firm distribution.
- Table S3 shows the primary industry and sector classification.

Sl. no.	Country name	country code	No. of firms	Total revenue	GDP (current US$) 2017
1	Afghanistan	AFG	34	365.6	20191764940.1602
2	Albania	ALB	64	1038.8	13025062195.7906
3	Algeria	DZA	135	16686.7	167555280113.181
4	Andorra	AND	8	464.53	3013387423.93509
5	Angola	AGO	132	27427.51	122123822333.591
6	Anguilla	AIA	7	0.279	-
7	Antigua and Barbuda	ATG	23	-	1510084750.74074
8	Argentina	ARG	1199	178835.56	642695864756.35
9	Armenia	ARM	56	1241.83	11527458565.7334
10	Aruba	ABW	19	238.7	2700558659.21788
11	Australia	AUS	11955	3894482.47199999	1330803227996.08
12	Austria	AUT	1500	495115.007	416835975862.194
13	Azerbaijan	AZE	83	32534.556	40865558912.3867
14	Bahamas, The	BHS	131	1036.314	12162100000
15	Bahrain	BHR	249	19700.806	35432686170.2128
16	Bangladesh	BGD	1514	29319.103	249723862487.361
17	Barbados	BRB	104	3406.7	4673500000
18	Belarus	BLR	128	8661.46	54726595249.1849
19	Belgium	BEL	2296	503814.929	494901708704.269
20	Benin	BEN	14	58.4	9246696923.66155
21	Bermuda	BMU	677	215629.304	-
22	Bhutan	BTN	25	376.8	2528007911.35353
23	Bolivia	BOL	108	11963.02	37508642257.5977
24	Bonaire	BES	1	-	-
25	Bosnia and Herzegovina	BIH	112	3998.21	18080118128.3854
26	Botswana	BWA	195	14360.19	17406565823.2986
27	Brazil	BRA	4059	1341430.151	2053594973992.61
28	British Virgin Islands	VGB	571	2646.326	-
29	Brunei	BRN	48	336	12128088999.9276
30	Bulgaria	BGR	578	28528.031	58220973782.7715
31	Burkina Faso	BFA	19	-	12322864244.9183
32	Burundi	BDI	8	137.14	3172416146.3921
33	Cote d'Ivoire	CIV	43	5911.3	38053610009.4172
34	Cambodia	KHM	159	2191.8	22177200511.5811
35	Cameroon	CMR	51	19648.33	34922782310.6416
36	Canada	CAN	15016	2789244.548	1646867220617.47
37	Cape Verde	CPV	9	173.5	1771235958.20125
38	Cayman Islands	CYM	482	17415.518	3570575151.26723
39	Central African Republic	CAF	4	-	2167501639.76783
40	Chad	TCD	10	-	9975692095.40855
41	Channel Islands	CHI	345	27779.03	-
42	Chile	CHL	1794	269727.3	277746457909.868
43	China	CHN	27633	11960538.449	12143491448186.1
44	Colombia	COL	1026	195727.402	311789874617.096
45	Comoros	COM	3	-	1068124329.86257
46	Congo (Brazzaville)	COG	49	1207.5	8701334800.21976
47	Congo Democratic Republic of	COD	46	1412.7	38019265625.856
48	Cook Islands	COK	5	-	-
49	Croatia	HRV	553	34162.332	55201417479.3925
50	Cuba	CUB	17	-	96851000000

51	Cyprus	CYP	538	58964.634	22141864998.8731
52	Czech Republic	CZE	1372	145468.25	215913545038.43
53	Denmark	DNK	1926	448918.056	329865537183.47
54	Djibouti	DJI	3	154.9	1844674434.50371
55	Dominica	DMA	11	-	496726248.518519
56	Dominican Republic	DOM	77	3072.6	75931656814.657
57	East Timor	TLS	8	-	2487269437.36822
58	Ecuador	ECU	172	7090.82	104295862000
59	Egypt	EGY	603	31545.301	235369129337.711
60	Eritrea	ERI	4	-	-
61	Estonia	EST	370	13558.41	26611651598.9453
62	Ethiopia	ETH	56	3458.8	81716326730.819
63	Falkland Islands	FLK	4	-	-
64	Fiji	FJI	53	1195.54	5270335184.70768
65	Finland	FIN	1920	340265.239	252301837573.029
66	France	FRA	8165	3448556.89	2586285406561.51
67	French Guiana	GUF	5	25.8	-
68	French Polynesia	PYF	7	69.7	-
69	Gabon	GAB	31	5759.1	14892609693.1667
70	Gambia The	GMB	8	-	1489464787.85603
71	Georgia	GEO	74	578.9	15081330942.4188
72	Germany	DEU	11574	4555411.97100001	3693204332229.78
73	Ghana	GHA	188	5918.08	58996776244.424
74	Gibraltar	GIB	76	1307.06	-
75	Greece	GRC	1985	113381.706	203085551429.132
76	Greenland	GRL	10	648.08	-
77	Grenada	GRD	5	70.4	1126882296.2963
78	Guadeloupe	GLP	5	-	-
79	Guinea	GIN	28	-	9915311049.15133
80	Guinea-Bissau	GNB	4	3080.4	1346841897.00437
81	Guyana	GUY	18	23	3555205811.13802
82	Haiti	HTI	11	49.3	8408252995.16143
83	Hong Kong	HKG	6725	1977552.828	341648103474.824
84	Hungary	HUN	764	90165.09	139761138102.757
85	Iceland	ISL	163	11710.79	24489493459.0074
86	India	IND	18502	1675896.42300001	2652551202555.27
87	Indonesia	IDN	10403	402879.001	1015423455783.28
88	Iran	IRN	286	40698.59	454012768723.589
89	Iraq	IRQ	93	1633.6	193158783783.784
90	Ireland	IRL	1755	614668.552999999	331430014003.047
91	Israel	ISR	2328	280344.674	353268411918.577
92	Italy	ITA	6998	1371163.738	1946570340341.13
93	Jamaica	JAM	191	7091.264	14781107821.7513
94	Japan	JPN	10775	8675056.03899996	4859950558538.97
95	Jordan	JOR	394	32611.639	40765867418.8999
96	Kazakhstan	KAZ	426	35509.18	162886867831.694
97	Kenya	KEN	348	21157.894	78757391333.0088
98	Kiribati	KIR	1	-	185572501.532802
99	Korea North	PRK	5	-	-
100	Korea South	KOR	4997	2626491.358	1530750923148.7
101	Kosovo	XKX	18	304.5	7227764976.79274
102	Kuwait	KWT	351	43292.112	119551599076.822
103	Kyrgyzstan	KGZ	35	452.2	7702934800.12836
104	Laos	LAO	50	848.4	16853087485.4118
105	Latvia	LVA	312	10913.567	30463302413.7289
106	Lebanon	LBN	166	6849.5	53393799668.325
107	Lesotho	LSO	13	4389.7	2578265355.71255
108	Liberia	LBR	112	-	3285455000
109	Libya	LBY	74	212.6	38115981878.5647
110	Liechtenstein	LIE	43	12177.55	-
111	Lithuania	LTU	524	25803.34	47544459558.9514
112	Luxembourg	LUX	653	206746.656	62316359824.1281
113	Macau	MAC	195	31353.683	50559431846.4989
114	Macedonia	MKD	66	1299.02	11279509013.9119

115	Madagascar	MDG	39	34.5	11465850504.0067
116	Malawi	MWI	53	5275.6	6303292264.18905
117	Malaysia	MYS	7936	408028.655000001	314707268049.991
118	Maldives	MDV	43	356.45	4865546025.86599
119	Mali	MLI	28	65.1	15339614406.6617
120	Malta	MLT	271	10381.83	12748803180.3035
121	Martinique	MTQ	5	-	-
122	Mauritania	MRT	17	727.3	4975432190.51025
123	Mauritius	MUS	369	11615.152	13259351418.4459
124	Mexico	MEX	2776	835615.566000002	1158071006809.62
125	Moldova	MDA	72	1166.43	9669759987.02633
126	Monaco	MCO	82	3054	6400946585.53076
127	Mongolia	MNG	118	-418.7	11433635875.9316
128	Montenegro	MNE	44	983.9	4844592066.71174
129	Morocco	MAR	231	52080.09	109708728848.535
130	Mozambique	MOZ	88	3827.3	12651912500.4128
131	Myanmar	MMR	116	669.2	66719084835.9898
132	Namibia	NAM	164	13079.7	13566192142.5835
133	Nauru	NRU	2	-	113880715.219337
134	Nepal	NPL	65	1420.2	24880266905.4961
135	Netherlands	NLD	4149	1755592.252	830572618849.829
136	Netherlands Antilles	ANT	58	8965.1	-
137	New Caledonia	NCL	15	13144.4	-
138	New Zealand	NZL	2155	396634.359999999	202590814084.991
139	Niger	NER	14	-	8119710126.32655
140	Nigeria	NGA	1294	33653.433	375745486520.656
141	Norway	NOR	3034	509958.53	399488897844.046
142	Oman	OMN	492	27061.932	70783875162.5488
143	Pakistan	PAK	1299	77117.213	304951818494.066
144	Palestinian Authority	PSE	35	1998.84	14498100000
145	Panama	PAN	150	15671.8	62283800000
146	Papua New Guinea	PNG	78	21203.77	22277692408.8879
147	Paraguay	PRY	67	1827.3	39008900331.6733
148	Peru	PER	813	105205.9	210702303186.432
149	Philippines	PHL	3255	242776.568	313619747740.186
150	Poland	POL	5339	490258.466	526371021088.561
151	Portugal	PRT	1400	157525.13	219308125506.737
152	Qatar	QAT	421	68578.38	166928571428.571
153	Reunion	REU	12	183.4	-
154	Romania	ROU	1333	85330.8500000001	211406933991.363
155	Russia	RUS	3913	1340757.433	1578624060588.26
156	Rwanda	RWA	34	261	9135454442.14013
157	Saint Kitts & Nevis	KNA	4	-	992007403.125926
158	Saint Lucia	LCA	20	96.3	1810139888.88889
159	Saint Vincent and The Grenadines	VCT	7	-	785222509.144563
160	San Marino	SMR	5	-	1632860040.56795
161	Saudi Arabia	SAU	1161	204125.646	688586133333.333
162	Senegal	SEN	47	2468.5	21081669870.0624
163	Serbia	SRB	360	11945.42	44120424391.86
164	Seychelles	SYC	28	284.1	1503168689.81984
165	Sierra Leone	SLE	23	-	3739577973.23943
166	Singapore	SGP	6354	1782500.835	338406474038.67
167	Slovakia	SVK	472	70136.38	95617670260.1145
168	Slovenia	SVN	384	29887.97	48455919386.0505
169	Solomon Islands	SLB	7	62.2	1321131090.73503
170	Somalia	SOM	10	-	7128000000
171	South Africa	ZAF	4417	833255.578	348871647962.321
172	Spain	ESP	4030	1093061.456	1314314164402.2
173	Sri Lanka	LKA	1502	29295.567	88019706803.834
174	Sudan	SDN	60	970.9	123053386001.137
175	Suriname	SUR	14	368.3	3068766109.75333
176	Swaziland	SWZ	33	552.2	4433664364.24725
177	Sweden	SWE	4978	894068.050000001	535607385506.432
178	Switzerland	CHE	3604	4488854.00900001	678965423322.021

179	Syria	SYR	68	1368.4	-
180	Taiwan	TWN	4189	1168175.276	-
181	Tajikistan	TJK	24	-	7157865188.25222
182	Tanzania	TZA	162	2866.22	53320625958.5628
183	Thailand	THA	5445	651112.432999999	455275517239.347
184	Togo	TGO	17	1200.9	4765866980.38429
185	Tonga	TON	7	-	430174168.740104
186	Trinidad and Tobago	TTO	127	9655.08	22250455018.8067
187	Tunisia	TUN	149	6308.4	39952095560.8829
188	Turkey	TUR	2488	404962.46	851549231502.615
189	Turkmenistan	TKM	10	-	37926285714.2857
190	Turks & Caicos Islands	TCA	13	-	962525840
191	Tuvalu	TUV	1	-	40620557.1335093
192	Uganda	UGA	91	879.62	25995031850.1545
193	Ukraine	UKR	734	47476.3630000001	112190355158.178
194	United Arab Emirates	ARE	2121	226929.05	382575085091.899
195	United Kingdom	GBR	24727	5795175.525	2637866340434.13
196	United States of America	USA	121475	29655109.9389996	19485393853000
197	Uruguay	URY	176	22385.08	56488991831.0239
198	Uzbekistan	UZB	52	6061.7	59159949231.4924
199	Vanuatu	VUT	11	9.46	849708342.698412
200	Vatican City	VAT	3	-	-
201	Venezuela	VEN	262	195097.56	-
202	Vietnam	VNM	2036	181079.663	223779865815.183
203	Western Samoa	WSM	33	-	841538412.998896
204	Yemen	YEM	52	609.664	26818703092.5852
205	Zambia	ZMB	138	7017.01	25868142076.7897
206	Zimbabwe	ZWE	162	4179.9	22813010116.1292
207	Unknown	-	32907	3757.57	-

TABLE S1: Countries and firm distribution. “-” in some fields indicates missing information

Sl. no.	Name of the sector	No. of firms
1	Communication Services	17491
2	Consumer Discretionary	35283
3	Consumer Staples	13044
4	Energy	9542
5	Financials	25953
6	Health Care	20979
7	Industrials	54819
8	Information Technology	34648
9	Materials	18019
10	Real Estate	7555
11	Utilities	7017
12	Unknown	193103

TABLE S2: Sectors and firm distribution

ID	Primary industry	Sector
1	Advertising	Communication Services
2	Aerospace and Defense	Industrials
3	Agricultural Products	Consumer Staples
4	Agricultural and Farm Machinery	Industrials
5	Air Freight and Logistics	Industrials
6	Airlines	Industrials
7	Airport Services	Industrials
8	Alternative Carriers	Communication Services
9	Aluminum	Materials
10	Apparel Retail	Consumer Discretionary
11	Apparel, Accessories and Luxury Goods	Consumer Discretionary
12	Application Software	Information Technology
13	Asset Management and Custody Banks	Financials
14	Auto Parts and Equipment	Consumer Discretionary
15	Automobile Manufacturers	Consumer Discretionary
16	Automotive Retail	Consumer Discretionary
17	Biotechnology	Health Care
18	Brewers	Consumer Staples
19	Broadcasting	Communication Services
20	Building Products	Industrials
21	Cable and Satellite	Communication Services
22	Casinos and Gaming	Consumer Discretionary
23	Coal and Consumable Fuels	Energy
24	Commercial Printing	Industrials
25	Commodity Chemicals	Materials
26	Communications Equipment	Information Technology
27	Computer and Electronics Retail	Consumer Discretionary
28	Construction Machinery and Heavy Trucks	Industrials
29	Construction Materials	Materials
30	Construction and Engineering	Industrials
31	Consumer Electronics	Consumer Discretionary
32	Consumer Finance	Financials
33	Copper	Materials
34	Data Processing and Outsourced Services	Information Technology
35	Department Stores	Consumer Discretionary
36	Distillers and Vintners	Consumer Staples
37	Distributors	Consumer Discretionary
38	Diversified Banks	Financials
39	Diversified Capital Markets	Financials
40	Diversified Chemicals	Materials
41	Diversified Metals and Mining	Materials
42	Diversified REITs	Real Estate
43	Diversified Real Estate Activities	Real Estate
44	Diversified Support Services	Industrials
45	Drug Retail	Consumer Staples
46	Education Services	Consumer Discretionary
47	Electric Utilities	Utilities
48	Electrical Components and Equipment	Industrials
49	Electronic Components	Information Technology
50	Electronic Equipment and Instruments	Information Technology
51	Electronic Manufacturing Services	Information Technology
52	Environmental and Facilities Services	Industrials
53	Fertilizers and Agricultural Chemicals	Materials
54	Financial Exchanges and Data	Financials
55	Food Distributors	Consumer Staples
56	Food Retail	Consumer Staples
57	Footwear	Consumer Discretionary
58	Forest Products	Materials
59	Gas Utilities	Utilities
60	General Merchandise Stores	Consumer Discretionary
61	Gold	Materials
62	Health Care Distributors	Health Care

63	Health Care Equipment	Health Care
64	Health Care Facilities	Health Care
65	Health Care REITs	Real Estate
66	Health Care Services	Health Care
67	Health Care Supplies	Health Care
68	Health Care Technology	Health Care
69	Heavy Electrical Equipment	Industrials
70	Highways and Railtracks	Industrials
71	Home Furnishings	Consumer Discretionary
72	Home Improvement Retail	Consumer Discretionary
73	Homebuilding	Consumer Discretionary
74	Homefurnishing Retail	Consumer Discretionary
75	Hotel and Resort REITs	Real Estate
76	Hotels, Resorts and Cruise Lines	Consumer Discretionary
77	Household Appliances	Consumer Discretionary
78	Household Products	Consumer Staples
79	Housewares and Specialties	Consumer Discretionary
80	Human Resource and Employment Services	Industrials
81	Hypermarkets and Super Centers	Consumer Staples
82	IT Consulting and Other Services	Information Technology
83	Independent Power Producers and Energy Traders	Utilities
84	Industrial Conglomerates	Industrials
85	Industrial Gases	Materials
86	Industrial Machinery	Industrials
87	Industrial REITs	Real Estate
88	Insurance Brokers	Financials
89	Integrated Oil and Gas	Energy
90	Integrated Telecommunication Services	Communication Services
91	Interactive Home Entertainment	Communication Services
92	Interactive Media and Services	Communication Services
93	Internet Services and Infrastructure	Information Technology
94	Internet and Direct Marketing Retail	Consumer Discretionary
95	Investment Banking and Brokerage	Financials
96	Leisure Facilities	Consumer Discretionary
97	Leisure Products	Consumer Discretionary
98	Life Sciences Tools and Services	Health Care
99	Life and Health Insurance	Financials
100	Managed Health Care	Health Care
101	Marine	Industrials
102	Marine Ports and Services	Industrials
103	Metal and Glass Containers	Materials
104	Mortgage REITs	Financials
105	Motorcycle Manufacturers	Consumer Discretionary
106	Movies and Entertainment	Communication Services
107	Multi-Sector Holdings	Financials
108	Multi-Utilities	Utilities
109	Multi-line Insurance	Financials
110	Office REITs	Real Estate
111	Office Services and Supplies	Industrials
112	Oil and Gas Drilling	Energy
113	Oil and Gas Equipment and Services	Energy
114	Oil and Gas Exploration and Production	Energy
115	Oil and Gas Refining and Marketing	Energy
116	Oil and Gas Storage and Transportation	Energy
117	Other Diversified Financial Services	Financials
118	Packaged Foods and Meats	Consumer Staples
119	Paper Packaging	Materials
120	Paper Products	Materials
121	Personal Products	Consumer Staples
122	Pharmaceuticals	Health Care
123	Precious Metals and Minerals	Materials
124	Property and Casualty Insurance	Financials
125	Publishing	Communication Services
126	Railroads	Industrials

127	Real Estate Development	Real Estate
128	Real Estate Operating Companies	Real Estate
129	Real Estate Services	Real Estate
130	Regional Banks	Financials
131	Reinsurance	Financials
132	Renewable Electricity	Utilities
133	Research and Consulting Services	Industrials
134	Residential REITs	Real Estate
135	Restaurants	Consumer Discretionary
136	Retail REITs	Real Estate
137	Security and Alarm Services	Industrials
138	Semiconductor Equipment	Information Technology
139	Semiconductors	Information Technology
140	Silver	Materials
141	Soft Drinks	Consumer Staples
142	Specialized Consumer Services	Consumer Discretionary
143	Specialized Finance	Financials
144	Specialized REITs	Real Estate
145	Specialty Chemicals	Materials
146	Specialty Stores	Consumer Discretionary
147	Steel	Materials
148	Systems Software	Information Technology
149	Technology Distributors	Information Technology
150	Technology Hardware, Storage and Peripherals	Information Technology
151	Textiles	Consumer Discretionary
152	Thrifts and Mortgage Finance	Financials
153	Tires and Rubber	Consumer Discretionary
154	Tobacco	Consumer Staples
155	Trading Companies and Distributors	Industrials
156	Trucking	Industrials
157	Water Utilities	Utilities
158	Wireless Telecommunication Services	Communication Services

TABLE S3: Primary industry and sector classification

We show a different color code of the nodes for the overexpression network of primary industries, which is shown in Fig 7 in main text. Here we use the node color according to their sector classification. From Fig 7 of main text and Fig S1, we observe the clustering among primary industries are formed based their sectors.

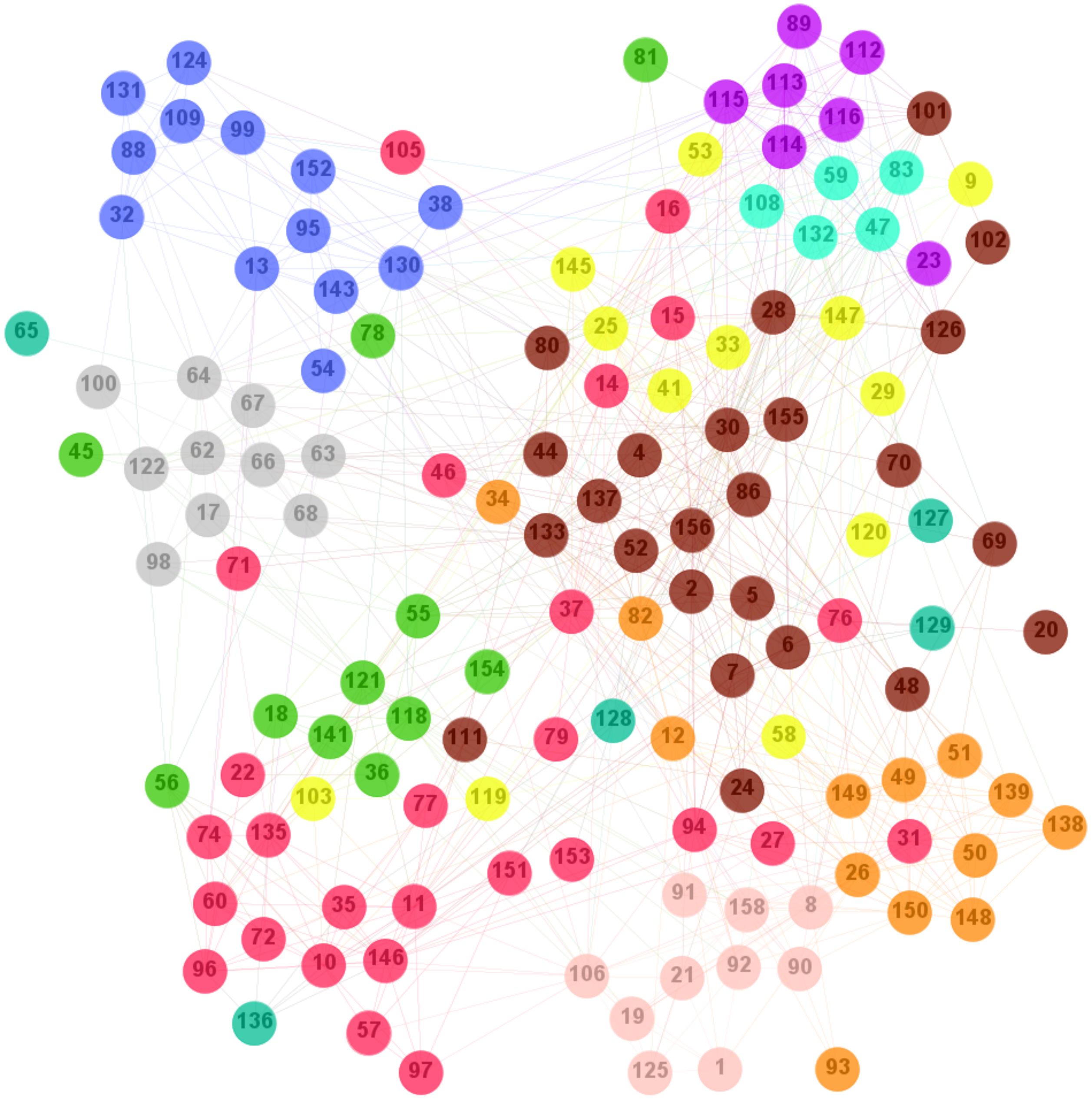

FIG. S1. **Overexpression network of primary industries. Different node color indicates different sectors. IDs of the nodes are given in Table S3.**
