# Supplementary material for: Testing “efficient supply chain propositions” using topological characterization of the global supply chain network
Source: PLoS One. 2020 Oct 1;15(10):e0239669. doi: 10.1371/journal.pone.0239669 (PMC7529254; doi:10.1371/journal.pone.0239669)
Supplement: Attachment — Submitted filename: Response to Reviewers.pdf [file pone.0239669.s003.pdf]

Reviewer #1: The Authors have made a series of substantial changes to the text, the result of which is a significantly improved manuscript. The Authors have reflected on all of my previous concerns and I accept their answers and corrections in most cases. However, I have three minor points which still need to be addressed. I refer to them with the numbers in my previous report.

Comment #1-1
6. In line 111: how do these statistics prove the goodness of the data?
・ I think I understand now, but the caption should also mention that total revenue on the vertical axis is measured for companies in the sample, not overall for the country.

Response to the comment:
We added the sentence, "The vertical axis is aggregated for firms of each country in the global supply chain data." to the caption of Fig. 1.

Comment #1-2
11.-12. In line 216 the Authors draw attention to a difference between their results and previous ones. There should be a discussion of this difference. Similarly, in line 220 there is a difference revealed with respect to many other networks. There should be a discussion about the reasons of this difference.
・ Still I feel that some discussion is missing, or at least clarifying the reasons why this discussion is not given here.

Response to the comment:
line 278-283 in the revised paper
We added the following explanation for the small SCC in the global supply chain network.

In Japan, there is a large SCC in the ownership network [1]. This is well known as cross-shareholding, or "keiretsu" (a set of companies with interlocking business relationships and shareholdings) in Japanese. Correspondingly, there is a large SCC in the Japanese supply chain. On the other hand, the cross-shareholdings are not as pronounced in the world's firms as in Japan. As a result, the SCC is small in the global supply chain network.

[1] Haruka Kato, Hitomi Sato, Yuichi Kichikawa, Hiroshi Iyetomi, Wataru Souma, and

Tsutomu Watanabe, Measurement of Value of Firms Based on Their Stock Ownership Relations, Book of Abstracts, the 8th Int'l Workshop on Complex Networks and their Applications (Lisbon, Portgal), 2019, pp. 450-452.

#1-3

17. In line 319 the Authors say that clustering is moderate. But compared to what?

・This should be indicated in the text as well.

Response to the comment:

Propositions S3:

However, the value of the clustering coefficient is not too high (<<1). The observed moderate clustering coefficient indicates that Proposition S3: It has a high clustering coefficient. is weakly valid.

We changed these sentences in line 410-413 in the revised paper to as follows.

However, the value of the clustering coefficient is equivalent to many other networks, such as the power grid, mobile phone calls, science collaboration, etc. The observed moderate clustering coefficient indicates that Proposition S3: It has a high clustering coefficient. is valid.

Reviewer #2: I think the authors have improved the quality of the manuscript by clarifying the aim of the paper. However, I have few remarks:

Comment #2-1
- the network is directed as the authors affirm, but the authors decided to apply some topological indicators using the directed network, while others have been applied using an undirected network. I think this choice must be justified. For instance, this is the case of community detection and clustering.

Response to the comment:
We have used an undirected version of the network for measuring the clustering coefficient and average nearest neighbor degree to show the presence of hierarchical structure and degree mixing property of the network as the basic structural properties. For community structure, we have used a directed version of the network where the links represent flow of goods from suppliers to customers.

At line 160 we added: We have used an undirected version of the network for measuring the clustering coefficient and average nearest neighbor degree to show the presence of hierarchical structure and degree mixing property of the network as the basic structural properties.

We modified line 286-288: We employ the map equation method [13] to discover the communities in the GWCC of the global supply chain network using the directed nature of the links. The directed links represent flow of goods from suppliers to customers.

Comment #2-2
- the authors use the undirected version of clustering coefficient, but I think they must cite the clustering they have used (Watts and Strogatz or Wasserman and Faust). Additionally, although the authors decided to stay in the undirected case, they have to mention that in literature clustering coefficients for directed case exists and cite them (see Fagiolo (2007), Clustering in Complex Directed Networks, Clemente, G.P. and Grassi, R. (2018) Directed clustering in weighted networks: a new perspective, Chaos, Solitons and Fractals.)

Response to the comment:

Line 151 in the revised paper: To calculate the clustering coefficient $C(k)$, we considered a simple undirected version of the network.

In the manuscript, we have added the following lines for the clustering coefficient at line 151-157:

The clustering coefficient C_i of i th node is defined as [1],

$$C_i = 2\, E_i / k_i(k_i - 1),$$

where E_i is the number of links between all the k_i neighbors of i. We have measured the clustering coefficient $<C(k)>$ averaged over the subset of nodes of degree k. Further one can measure more complex clustering coefficient considering the directed nature of links [2]. However, we only calculate the simple undirected clustering coefficient to show the basic structural property of the network.

[1] D. J. Watts and Steven Strogatz (June 1998). "Collective dynamics of 'small-world' networks". Nature. 393 (6684): 440–442

[2] Fagiolo (2007), Clustering in Complex Directed Networks, Clemente, G.P. and Grassi, R. (2018) Directed clustering in weighted networks: a new perspective, Chaos, Solitons and Fractals.

Reviewer #3: Most points have been addressed by the authors, and the manuscript has a much clearer argumentation. However, one main concern that I had with the first version of the manuscript is still not sufficiently addressed.

Comment #3-1

This main concern is the lack of conceptual integration of the two major ambitions of the manuscript (1: the bow-tie structure and 2: testing nine propositions of Hearnshaw et al.) The authors managed to integrate testing Hearnshaw et al. into the manuscript much better. But it is still not clear how the bow-tie finding integrates with this ambition. Does the bow-tie structure add a new topological requirement to having an efficient supply chain network? In that case, the bow-tie structure is a conceptual addition to Hearnshaw et al. Or is the bow-tie merely a consequence of Hearnshaw et al.'s requirements that they have not recognized? In that case the bow-tie structure is an empirical discovery that does not alter Hearnshaw et al.'s conceptualization.

These concerns are critical, as the own contribution of the manuscript is not clear. The ambition to test Hearnshaw et al. is clear now already in the abstract, but there is no mention about conclusions: what does this manuscript contribute? This is connected with the ambiguous language of "verifying". While I recognize that there are multiple methodological traditions in science, I would believe that there is the broadest agreement that "verification" is never possible in the empirical sciences, and we should think about "testing" instead. A theory will never be verified, but rather tested, and found support for. I would strongly urge the authors to re-phrase "verification" for testing, with an indication of what aspects of the theory from Hearnshaw et al. they found support for. This could help clarify the contribution of the manuscript as well. If I was the author, I would explore ways in which the novelty of the contribution can be identified, and highlighted already in the abstract.

Response to the comment:

I appreciate the excellent suggestion. I agree to re-phrase "verification" for "testing." However, to my knowledge, in the natural sciences, the word "verify" is sometimes used in the same sense as "test." For example, "experimental verification" is found in many literature.

We added the following sentences after Line 383 in the revised paper and changed the abstract and conclusions.

Original propositions of Hearnshaw S1 just mentioned a short characteristics path

length. As described in the discussion of Table 4, the upstream or downstream firms are located only a few steps away from the GSCC. The short path from the GSCC to IN and OUT was only discovered by analyzing the bow-tie structure. Therefore, the short path length in the bow-tie structure is a conceptual addition to the original propositions of Hearnshaw.

Comment #3-2

As a more minor point, the network overexpression graphs for the country and industry level are much more readable, but their integration into the ambitions of the manuscript is still not clear. What aspect of Hearnshaw et al. do these talk to? I would not include almost two full page graphs that are part of only a sideline discussion, receiving only a passing mention in the text.

Response to the comment:

We added the following sentences in Line 455-463 in the revised paper.

The over-expression of countries and sectors in the large communities shown in Table 5 allows us to characterize community formation by two factors. One is the over-expression of countries in the large communities shown in Fig. 6. The other is the over-expression network of sectors shown in Figure 7. In these figures, a country tends to form communities in neighboring countries, and a sector tends to form communities in the same industry. This result suggests that community formation is due to multiple factors, and the over-expression networks in Fig.6 and Fig.7 provide support for the appropriateness of the overlapping boundary of community formation in Proposition S7.
